# Horizontal extent of the urban heat dome flow

**DOI:** 10.1038/s41598-017-09917-4

**Published:** 2017-09-15

**Authors:** Yifan Fan, Yuguo Li, Adrian Bejan, Yi Wang, Xinyan Yang

**Affiliations:** 1 Department of Mechanical Engineering, The University of Hong Kong, Pok Fu Lam Road, Hong Kong, SAR China; 2 Department of Mechanical Engineering and Materials Science, Duke University, Durham, USA; 3 School of Environmental and Municipal Engineering, Xi’an University of Architecture and Technology, Xi’an, Shaanxi 710055 P.R. China

## Abstract

Urban heat dome flow, which is also referred to as urban heat island circulation, is important for urban ventilation and pollutant transport between adjacent cities when the background wind is weak or absent. A “dome-shaped” profile can form at the upper boundary of the urban heat island circulation. The horizontal extent of the heat dome is an important parameter for estimating the size of the area it influences. This study reviews the existing data on the horizontal extent of the urban heat dome flow, as determined by using either field measurements or numerical simulations. A simple energy balance model is applied to obtain the maximum horizontal extent of a single heat dome over the urban area, which is found to be approximately 1.5 to 3.5 times the diameter of the city’s urban area at night. A linearized model is also re-analysed to calculate the horizontal extent of the urban heat dome flow. This analysis supports the results from the energy balance model. During daytime, the horizontal extent of the urban heat dome flow is found to be about 2.0 to 3.3 times the urban area’s diameter, as influenced by the convective turbulent plumes in the rural area.

## Introduction

**Figure 1:**
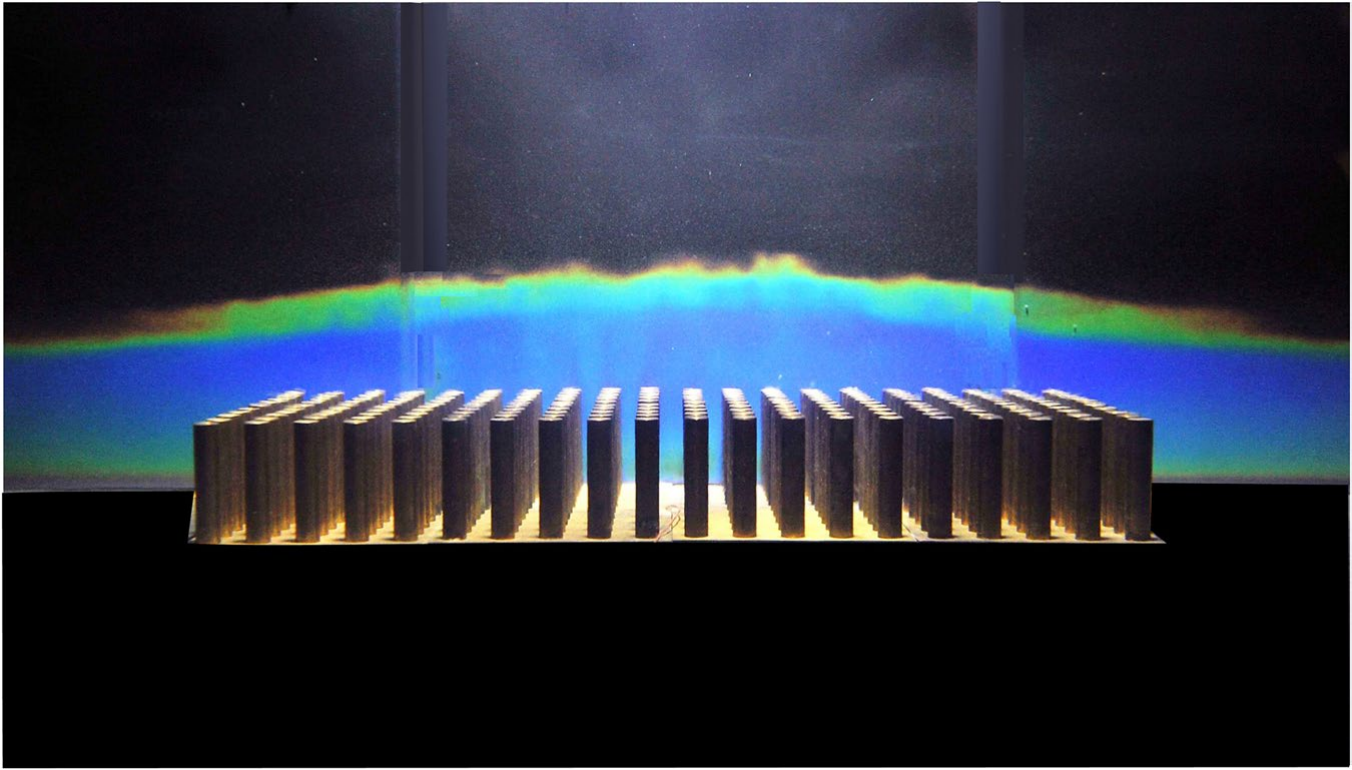
The dome-shaped upper thermal boundary between the mixing layer and the inversion layer, as visualized with thermochromic liquid crystals.

Urban heat dome flow, which is also called urban heat island–induced circulation^1–3^, is induced by the differences between urban and rural temperatures under conditions of inversion with calm background weather. The main factors that induce these mean circulating flows are the horizontal pressure gradients between urban and rural areas and inhomogeneous plumes impinging on the inversion layer. Urban heat domes are characterized by convergent inflows at the atmosphere’s lower level, upward flows in the form of turbulent plumes^4^ over the urban area, divergent outflow in the atmosphere’s upper level, and a “dome-shaped” upper boundary (See Fig. 1) at the interface between the inversion layer and the divergent outflow region^5^.

Under stable stratification and calm background conditions, the dispersion of pollutants and heat within and over the urban canopy layers (under which most human activities take place) depends mainly o n the urban heat dome flow^6–9^. The mixing height (i.e., the vertical extent) of the dome has been extensively examined, both quantitatively^10–12^ and theoretically^13^. However, the horizontal extent of the urban heat dome flow has received relatively little attention from researchers. Still, several studies have been done in which the dome horizontal extent (dome diameter) can be estimated, based on the numerical simulations and field measurements, which are summarized in Table 1.

**Table 1:** A summary of dome horizontal extent measurements, as extracted from existing studies using numerical models and field measurements.

Dome horizontal extent	Reference	Measured time	Further remarks
About 3*D*.	Ryu *et al*.^14^	17:30 Local Standard Time (LST)	Computational fluid dynamics (CFD) simulation; *D* is about 20 km.
About 3*D* to 4*D*.	Lemonsu and Masson^15^	Afternoon	CFD simulation; *D* is about 25-30 km.
About 2*D* to 2.6*D*.	Eliasson and Holmer^16^	Night-time	Field measurements; *D* is about 10 km.
About 2*D* (3.9*D*)	Ganbat *et al*.^17^	Daytime at 13:00 (19:00) LST.	*D* is about 20 km.
About 2*D*.	Hidalgo *et al*.^18^	Daytime (12:00–18:00)	CFD simulation (Toulouse, France).
About 2*D* to 3*D*.	Hidalgo *et al*.^19^	Daytime (12:00–18:00)	Field measurement (Toulouse, France).

*D* is the accordingly calculated urban diameter.

The mechanisms and factors governing the domes’ horizontal extent were not discussed in these previous studies, and no physical model was proposed to explain and predict the domes’ horizontal extent or their diameters.

When cities are grouped together to form a city cluster, each city may generate its own heat dome. Under calm background conditions, the pollutants may be transported between adjacent cities through the dome flow, and thus cause regional air pollution. Various large city clusters have arisen in Asia, such as the Beijing-Tianjin-Hebei region, the Yangtze River delta region, and the Pearl River delta region in China. The mechanisms and characteristics of the urban heat dome flows are important to consider for understanding pollutant dispersion, urban heat removal, and the transport of regional pollutants between adjacent cities under calm, stably stratified background environments.

It should be noted that the urban heat dome flow reaches quasi-steady state about 4 hours after sunrise^20,21^ and about 4–6 hour after sunset^16,22^. The urban heat dome flow is in the transition state around sunset or sunrise time^14^, which is not considered in this study. Therefore, in this study, the daytime refers to the time slot between the time about 4 hours after sunrise and the time before the sunset, when the daytime urban heat dome flow is in the quasi-steady state. The night-time refers to the time slot between the time about 4–6 hour after sunset and the time before the sunrise, when the night-time urban heat dome flow is in the quasi-steady state.

The energy balance model and linearized model are presented in Section 2.1 and Section 2.2 respectively. The horizontal extent of the urban heat dome during daytime is determined in Section 2.3. The implication of the results, the limitation of the models and the relationship between the dryland warming, proposed by Huang *et al*.^23,24^, and the urban heat dome are discussed in Section 3.

## Results

### Energy balance model

#### Model description

As first proposed by Fan *et al*.^5^, an urban dome’s horizontal extent may be determined from the energy balance model, assuming a steady state of flow in the urban heat dome. With a further assumption that any turbulent exchange at the dome perimeter and any energy dissipation are neglected, the energy balance equation for the dome flow at night-time can be estimated with Equation (1).

1 $${\iint }_{{A}_{pu}}{H}_{u}d{A}_{pu}={\iint }_{{A}_{pr}}-{H}_{r}d{A}_{pr}$$

where *H*_*u*_ and *H*_*r*_ are the heat flux in the urban area and the rural area, respectively, *A*_*pu*_ and *A*_*pr*_ are the urban plenary area (with the areas of building roofs included) and the rural plenary area, respectively. Lateral entrainment in the dome is ignored, because the dome is assumed to be in a steady state, without further growth.

**Figure 2:**
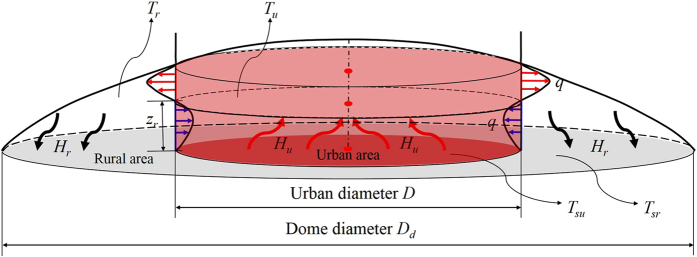
Illustration of the energy balance model of urban heat dome flow. *D* is the urban diameter, and *D*_*d*_ is the dome diameter. *T*_*su*_ and *T*_*sr*_ are the average surface temperatures in the urban area and in the rural area, respectively. *T*_*u*_ and *T*_*r*_ are the average air temperatures in the urban area and in the rural area, respectively. *z*_*r*_ is the reverse height, i.e., the height of the interface between the convergent inflow and the divergent outflow. *q* is the volume flux of the inflow and the outflow. A steady state is assumed, so that the volumes of flux for the inflow and the outflow are the same, based on the principle of mass conservation and the Boussinesq approximation.

The energy for maintaining the urban heat dome flow is fed by the positive heat flux (from the surface to the urban heat dome) in the urban area, i.e., the left side of Equation (1). The air is heated up over the urban area, and is then transported to the rural area by the urban heat dome flow. The surface temperature of the rural area decreases at night-time, due to radiative cooling. Therefore, where the urban heat dome extends over the rural area, its heat is transported to the rural surface. This effect is the mechanism for the formation of stable stratification in the rural boundary layer, i.e., the right side of Equation (1). This process is illustrated in Fig. 2.

The governing equations for determining the energy balances for the temperatures of the urban air, the urban surface, the rural air, and the rural surface are given in Equations (2)–(5), respectively.

2 $$\rho {c}_{p}q({T}_{r}-{T}_{u})+{h}_{c}{A}_{tu}({T}_{su}-{T}_{u})+{q}_{a}{A}_{pu}=0$$

3 $${A}_{tu}{\lambda }_{M}\sum _{\begin{array}{c}j=-\infty \\ j\ne 0\end{array}}^{+\infty }\frac{1+i}{{d}_{ju}}{\tilde{T}}_{su}(j\omega )\exp (ij\omega t)=(1-{\alpha }_{u}){A}_{pu}{q}_{sol}-{h}_{c}{A}_{tu}({T}_{su}-{T}_{u})-{A}_{tu}{q}_{rad,u}$$

4 $$\rho {c}_{p}q({T}_{u}-{T}_{r})+{h}_{c}{A}_{pr}({T}_{sr}-{T}_{r})=0$$

5 $${A}_{pr}{\lambda }_{M}\sum _{\begin{array}{c}j=-\infty \\ j\ne 0\end{array}}^{+\infty }\frac{1+i}{{d}_{jr}}{\tilde{T}}_{sr}(j\omega )\exp (ij\omega t)=(1-{\alpha }_{r}){A}_{pr}{q}_{sol}-{h}_{c}{A}_{pr}({T}_{sr}-{T}_{r})-{A}_{pr}{q}_{rad,r}$$

where *ρ* and *c*_*p*_ are the air density and the heat capacity, respectively. *h*_*c*_ is the convective heat-transfer coefficient. *T*_*su*_ and *T*_*sr*_ are the average surface temperatures in the urban area and the rural area, respectively. *T*_*u*_ and *T*_*r*_ are the average air temperatures in the urban area and the rural area, respectively. For a circular-shaped city, the dome footprint is also circular. ${A}_{pu}=\pi {D}^{2}/4$ and ${A}_{pr}=\pi {D}_{d}^{2}/4-\pi {D}^{2}/4$ are the urban plenary area (with the areas of building roofs included) and the rural plenary area, respectively. ${A}_{tu}={A}_{pu}+{A}_{wu}$ is the total surface of the urban area. *A*_*wu*_ is the total area of all the building walls (with roof areas not included). *q* is the volume flux of the inflow and the outflow. *q*_*a*_, *q*_*sol*_, *q*_*rad,u*_ and *q*_*rad,r*_ are the anthropogenic heat flux in the urban area, the solar radiative heat flux, the long wave radiative heat flux in the urban area, and the long wave radiative heat flux in the rural area, respectively. *α*_*u*_ and *α*_*r*_ are the average albedo in the urban area and the rural area, respectively. *λ*_*M*_ is the thermal conductivity of the surface material. *d*_*jru*_ and ${\tilde{T}}_{su}(j\omega )$ are the temperature penetration depth and the temperature fluctuation amplitude for the corresponding angular frequency waves in the urban area, respectively. *d*_*jr*_ and ${\tilde{T}}_{sr}(j\omega )$ are the temperature penetration depth and the temperature fluctuation amplitude for the corresponding angular frequency waves in the rural area, respectively. *i* is an imaginary number. *ω* is the angular frequency.

To simplify the governing equations, the following parameters are defined, following Yang^25^. ${f}_{w}={A}_{wu}/{A}_{pu}$ is the ratio of the total building wall surface area to the plenary surface in the urban area. $\lambda ={h}_{c}{A}_{pu}/(\rho {c}_{p}q)$ is the convective heat transfer number. ${\lambda }_{sky,u}={h}_{rad,u}{A}_{pu}/(\rho {c}_{p}q)$ and ${\lambda }_{sky,r}={h}_{rad,r}{A}_{pu}/(\rho {c}_{p}q)$ are the sky radiation heat transfer numbers for the urban area and the rural area, respectively, where ${h}_{rad,u}={h}_{rad}{F}_{svf}$ and ${h}_{rad,r}={h}_{rad}$ are the radiative heat-transfer coefficients for the urban area and the rural area, respectively. *h*_*rad*_ is the radiative heat-transfer coefficient. ${q}_{rad,r}={h}_{rad,r}({T}_{sr}-{T}_{sky})$ and ${q}_{rad,u}={h}_{rad,u}({T}_{su}-{T}_{sky})$. *F*_*svf*_ is the average sky view factor in the urban area. *T*_*sky*_ is the background sky temperature. Definitions are given for the increases in temperature, ${\rm{\Delta }}{T}_{a}={q}_{a}{A}_{pu}/(\rho {c}_{p}q)$, ${\rm{\Delta }}{T}_{sol,u}=(1-{\alpha }_{u}){q}_{sol}{A}_{pu}/(\rho {c}_{p}q)$, ${\rm{\Delta }}{T}_{u}={H}_{u}{A}_{pu}/(\rho {c}_{p}q)$, and ${\rm{\Delta }}{T}_{sol,r}=(1-{\alpha }_{r}){q}_{sol}{A}_{pu}/(\rho {c}_{p}q)$ that result from the anthropogenic heat, the solar radiation heat flux in the urban area, the sensible heat flux in the urban area, and the solar radiation heat flux in the rural area, respectively. The sensible heat flux in the urban area and the rural area can be expressed as ${H}_{u}=[{h}_{c}{A}_{tu}({T}_{su}-{T}_{u})+{q}_{a}{A}_{pu}]/{A}_{pu}$ and ${H}_{r}={h}_{c}({T}_{sr}-{T}_{r})$, respectively.

The steady state governing equations for the urban air, urban surface, rural air, and rural surface can be simplified as Equations (6)–(9), respectively, after the above-defined parameters are integrated into Equations (2)–(5).

6 $$\lambda (1+{f}_{w}){T}_{su}+{T}_{r}+{\rm{\Delta }}{T}_{a}=[1+\lambda (1+{f}_{w})]{T}_{u}$$

7 $$\frac{1}{1+{f}_{w}}{\rm{\Delta }}{T}_{sol,u}=(\lambda +{\lambda }_{sky,u}){T}_{su}-(\lambda {T}_{u}+{\lambda }_{sky,u}{T}_{sky})$$

8 $$\lambda {A}_{pr}{T}_{sr}+{A}_{pu}{T}_{u}=({A}_{pu}+\lambda {A}_{pr}){T}_{r}$$

9 $${\rm{\Delta }}{T}_{sol,r}=(\lambda +{\lambda }_{sky,r}){T}_{sr}-(\lambda {T}_{r}+{\lambda }_{sky,r}{T}_{sky})$$

Equations (6–9) can be solved as

$${T}_{u}=\frac{(\lambda +{\lambda }_{sky,u})({\rm{\Delta }}{T}_{a}-{\rm{\Delta }}{T}_{u})+\lambda [(1+{f}_{w}){\lambda }_{sky,u}{T}_{sky}+{\rm{\Delta }}{T}_{sol,u}]}{(1+{f}_{w}){\lambda }_{sky,u}\lambda }$$

$${T}_{su}=\frac{{\rm{\Delta }}{T}_{a}-{\rm{\Delta }}{T}_{u}+(1+{f}_{w}){\lambda }_{sky,u}{T}_{sky}+{\rm{\Delta }}{T}_{sol,u}}{(1+{f}_{w}){\lambda }_{sky,u}}$$

$${T}_{r}=\frac{({\lambda }_{sky,u}+\lambda ){\rm{\Delta }}{T}_{a}+\lambda [(1+{f}_{w}){\lambda }_{sky,u}{T}_{sky}+{\rm{\Delta }}{T}_{sol,u}]-[\lambda +{\lambda }_{sky,u}\lambda (1+{f}_{w})+{\lambda }_{sky,u}]{\rm{\Delta }}{T}_{u}}{(1+{f}_{w}){\lambda }_{sky,u}\lambda }$$

$$\begin{array}{c}{T}_{sr}=\frac{({\lambda }_{sky,u}+\lambda ){\rm{\Delta }}{T}_{a}+\lambda [(1+{f}_{w}){\lambda }_{sky,u}{T}_{sky}+{\rm{\Delta }}{T}_{sol,u}]+{\lambda }_{sky,u}(1+{f}_{w})({\lambda }_{sky,r}{T}_{sky}+{\rm{\Delta }}{T}_{sol,r})}{(1+{f}_{w})({\lambda }_{sky,r}+\lambda ){\lambda }_{sky,u}}\\ \quad \,\,\,\,\,\,-\frac{[\lambda +{\lambda }_{sky,u}\lambda (1+{f}_{w})+{\lambda }_{sky,u}]{\rm{\Delta }}{T}_{u}}{(1+{f}_{w})({\lambda }_{sky,r}+\lambda ){\lambda }_{sky,u}}\end{array}$$

$$\frac{{D}_{d}}{D}=\sqrt{\begin{array}{c}\frac{({\lambda }_{sky,u}+\lambda ){\rm{\Delta }}{T}_{a}{\lambda }_{sky,r}+\lambda {\lambda }_{sky,r}{\rm{\Delta }}{T}_{sol,u}-(1+{f}_{w})\lambda {\lambda }_{sky,u}{\rm{\Delta }}{T}_{sol,r}}{({\lambda }_{sky,u}+\lambda ){\rm{\Delta }}{T}_{a}{\lambda }_{sky,r}+\lambda {\lambda }_{sky,r}{\rm{\Delta }}{T}_{sol,u}-(1+{f}_{w})\lambda {\lambda }_{sky,u}{\rm{\Delta }}{T}_{sol,r}-{\lambda }_{sky,r}{\rm{\Delta }}{T}_{u}[\lambda +{\lambda }_{sky,u}+\lambda {\lambda }_{sky,u}(1+{f}_{w})]}+\\ \frac{{\rm{\Delta }}{T}_{u}\{{\lambda }_{sky,u}[{\lambda }_{sky,r}{f}_{w}+(1+{f}_{w})\lambda ]-\lambda {\lambda }_{sky,r}[1+{\lambda }_{sky,u}(1+{f}_{w})]\}}{({\lambda }_{sky,u}+\lambda ){\rm{\Delta }}{T}_{a}{\lambda }_{sky,r}+\lambda {\lambda }_{sky,r}{\rm{\Delta }}{T}_{sol,u}-(1+{f}_{w})\lambda {\lambda }_{sky,u}{\rm{\Delta }}{T}_{sol,r}-{\lambda }_{sky,r}{\rm{\Delta }}{T}_{u}[\lambda +{\lambda }_{sky,u}+\lambda {\lambda }_{sky,u}(1+{f}_{w})]}\end{array}}$$

**Figure 3:**
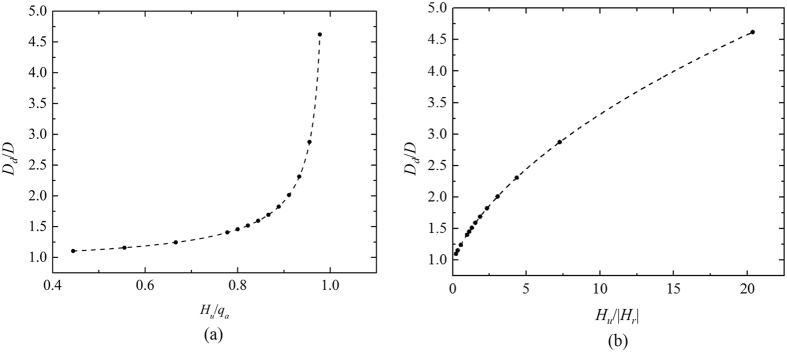
Results for the dome diameter, with *D* = 40 km, *N = *0.015 s^−1^, and *f*_*w*_ = 0.8 fixed. (**a**) The ratio of the dome diameter to the urban diameter (*D*_*d*_/*D*), which changes with the ratio of the urban sensitive heat flux to the anthropogenic heat flux. (**b**) The ratio of the dome diameter to the urban diameter (*D*_*d*_/*D*), which changes with the ratio of the urban sensitive heat flux to the absolute values of the rural sensible heat flux.

In the at-night condition, the terms containing ${\rm{\Delta }}{T}_{sol,r}$ and ${\rm{\Delta }}{T}_{sol,u}$ should be zero. The ratio of the dome diameter to the urban diameter can be simplified as ${D}_{d}/D=\sqrt{1+(B-A){\rm{\Delta }}{T}_{u}/(C{\rm{\Delta }}{T}_{a}-A{\rm{\Delta }}{T}_{u})}=$ $\sqrt{1+(B-A){H}_{u}/(C{q}_{a}-A{H}_{u})}$, where $A={\lambda }_{sky,r}[\lambda +{\lambda }_{sky,u}+\lambda {\lambda }_{sky,u}(1+{f}_{w})]$, .., and $C={\lambda }_{sky,r}(\lambda +{\lambda }_{sky,u})$. As can be seen, the ratio *D*_*d*_/*D* is related to sensible heat flux in the urban area and the anthropogenic heat flux. Because the urban sensible heat flux and the anthropogenic heat flux influence the air temperature both in the urban area and in the rural area, the sensible heat flux in the rural area is also influenced and thus the rural sensible heat flux is also an indicator for the ratio *D*_*d*_/*D*, which is shown in Fig. 3(b).

#### Experiment design on the model

The typical value for the anthropogenic heat flux at night, *q*_*a*_ = 45 W m^−2^, can be found in Sailor *et al*.^26^. For the city diameter, *D* = 40 km is used. According to Yang^25^, the albedo in the urban and the rural area, the sky temperature, and the ratios of the building walls are set as *α*_*u*_ = 0.2, *α*_*r*_ = 0.3, *T*_*sky*_ = 293.15 K, and ${f}_{w}={A}_{wu}/{A}_{pu}$ = 0.8, respectively. For the average sky view factor in the urban area, *F*_*svf*_ = 0.12 is used, based on Yang and Li^27^. The typical buoyancy frequency is given as *N* = 0.015 s^−1^ ^20,28^. The typical radiative heat-transfer coefficient, *h*_*rad*_ = 5 W m^−2^ K^−1^, and the typical convective heat transfer-coefficient, *h*_*c*_ = 18 W m^−2^ K^−1^, are obtained from Yang and Li^29^. The volume flux of the urban heat dome flow is estimated by $q={\int }_{0}^{{z}_{r}}\pi D{u}_{D}{\rm{d}}z$, where *u*_*D*_ is the horizontal velocity of the urban heat dome flow, and *z*_*r*_ is the reverse height of the urban heat dome flow. The velocity and the reverse height, *z*_*r*_, can be calculated by ${u}_{D}={[g\beta D{H}_{u}/\rho {c}_{p}]}^{1/3}$ and ${z}_{r}=1.03\,{u}_{D}/N$, respectively, as given in Lu *et al*.^10^.

#### Results of the energy balance model

**Figure 4:**
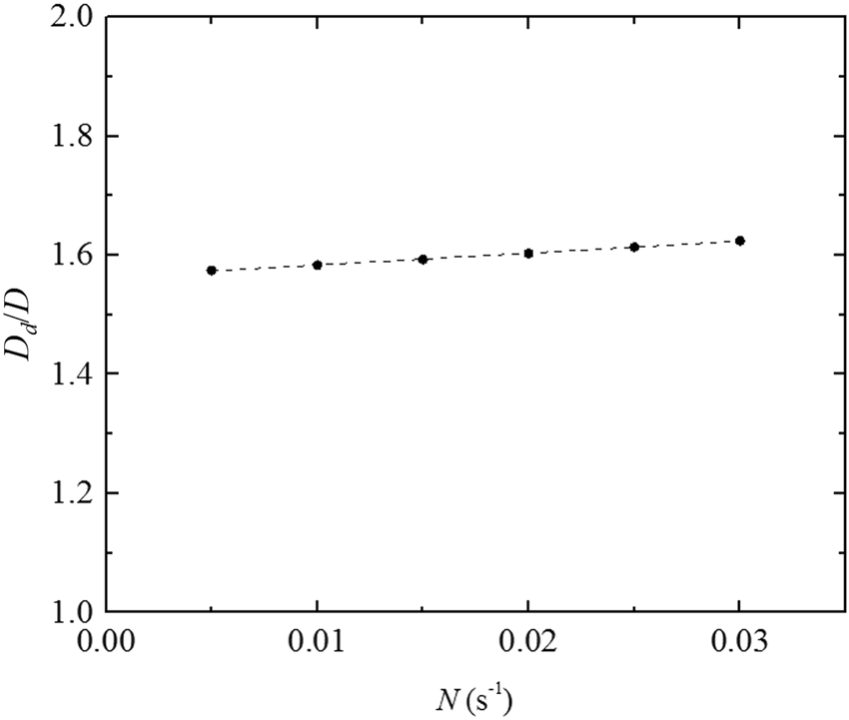
The ratio of the dome diameter to the urban diameter (*D*_*d*_/*D*), which changes with the background buoyancy.

**Figure 5:**
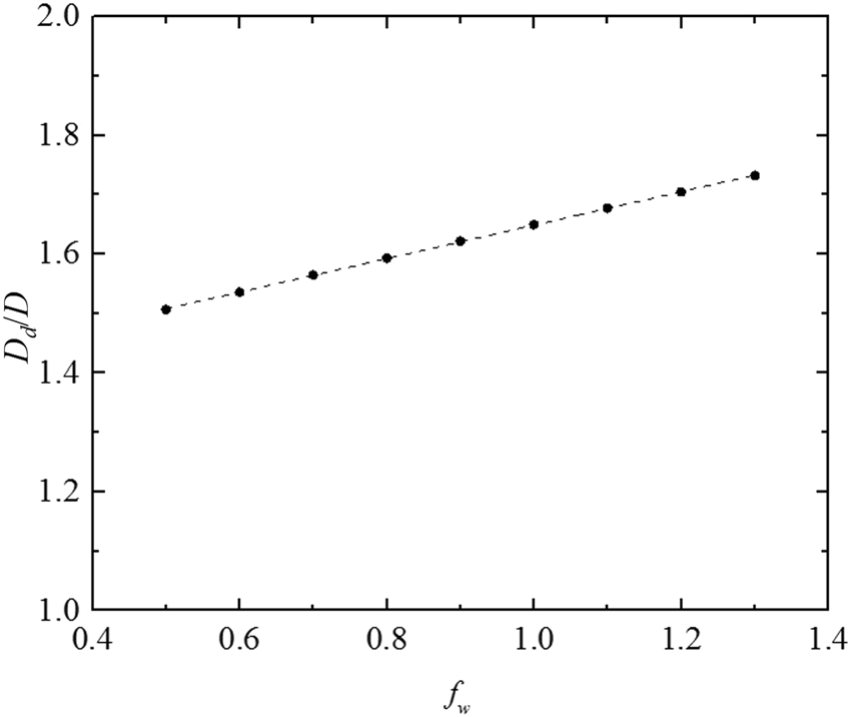
The ratio of the dome diameter to the urban diameter (*D*_*d*_/*D*), which changes with the ratio of the building walls.

The results are shown in Figs 3–5. The results in Fig. 3 are obtained based on the solution ${D}_{d}/D=\sqrt{1+(B-A){\rm{\Delta }}{T}_{u}/(C{\rm{\Delta }}{T}_{a}-A{\rm{\Delta }}{T}_{u})}=\sqrt{1+(B-A){H}_{u}/(C{q}_{a}-A{H}_{u})}$ with parameters urban diameter *D* = 40 km, background buoyancy frequency *N* = 0.015 s^−1^, and the ratio of the total building wall surface area to the plenary surface in the urban area *f*_*w*_ = 0.8 being fixed. The values of other parameters used in the model are described in detail in Section 2.1.2. The results in Figs 4 and 5 are obtained based on the same solution as used in Fig. 3, but the values of the parameters used are slightly different (*D* = 40 km, *H*_*u*_ = 38 W m^−2^, *f*_*w*_ = 0.8 and all other parameters described in Section 2.1.2 being fixed are used in Fig. 4. *D* = 40 km, *H*_*u*_ = 38 W m^−2^, *N* = 0.015 s^−1^ and all other parameters described in Section 2.1.2 being fixed are used in Fig. 5).

The solid circles in Figs 3–5 are calculated by the energy balance model for different cases (with changes in one of the parameters). The dashed lines in Figs 3–5 are fitted based on results calculated by the energy balance model, and the fitted results are as follows: ${D}_{d}/D=\sqrt{1+\frac{0.265{H}_{u}/{q}_{a}}{1-{H}_{u}/{q}_{a}}}$, ${D}_{d}/D=\sqrt{1+{H}_{u}/|{H}_{r}|}$, ${D}_{d}/D=2.00\,N+1.56$, and ${D}_{d}/D=0.28{f}_{w}+1.37$, respectively.

**Figure 6:**
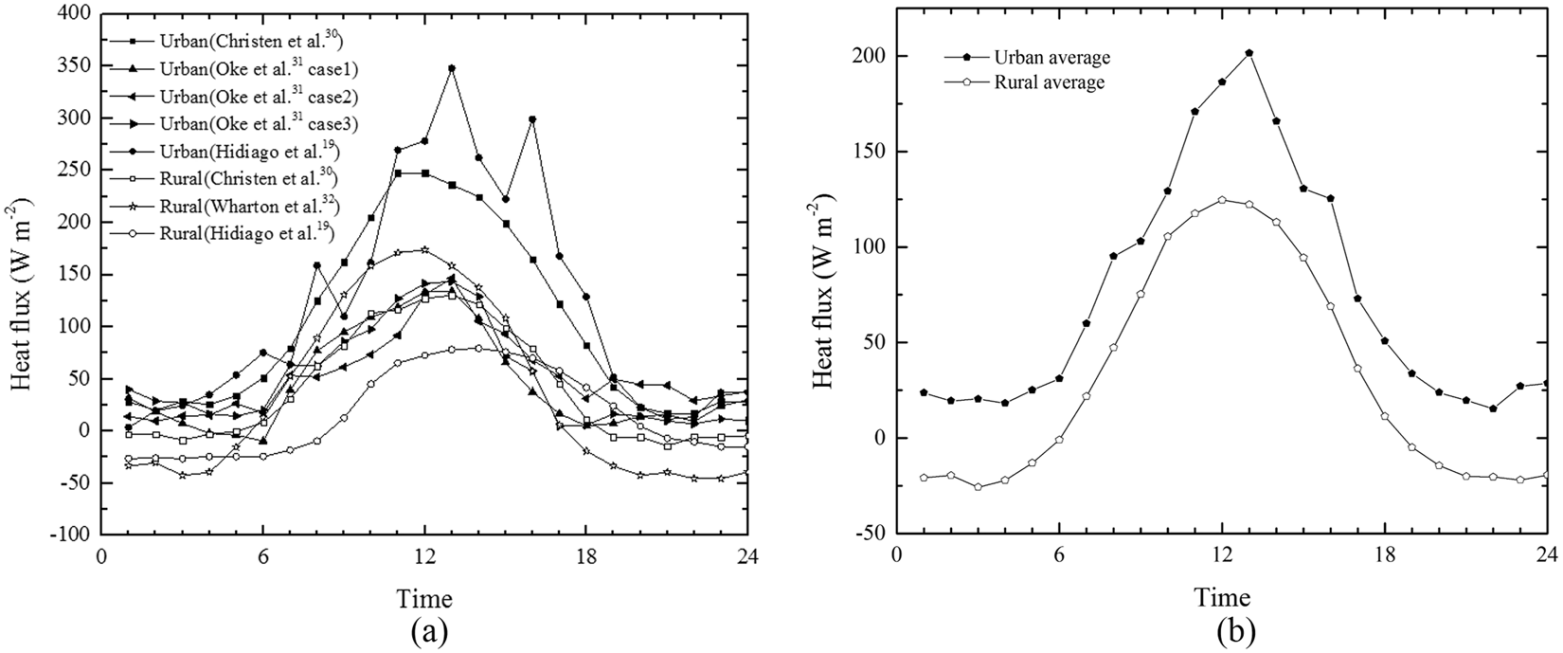
Typical profiles of sensible heat flux in urban areas and rural areas, as reported in the literature. The solid symbols represent sensible heat flux in urban areas, and the hollow symbols represent sensible heat flux in the rural areas. (**a**) The data are drawn from Christen *et al*.^30^, Oke *et al*.^31^, Hidalgo *et al*.^19^, and Wharton *et al*.^32^. The data given by Hidalgo *et al*.^19^ were originally half-hour averaged, and therefore two successive sets of data were averaged in this figure to get one-hour averaged data, to compare with the other results. (**b**) The average sensible heat flux is calculated based on the data given in (**a**).

The data from Christen *et al*.^30^ and Hidalgo *et al*.^19^ are used to obtain the typical ratios of night-time sensible heat fluxes *H*_*u*_/|*H*_*r*_|, because these studies include measures of both the urban sensible heat flux and the adjacent rural sensible heat flux in their estimates of sensible heat flux. The typical values of sensible heat flux obtained from different field measurements are summarized in Fig. 6.

These ratios are calculated using the data from Christen *et al*.^30^ and Hidalgo *et al*.^19^, and are shown in Fig. 6. In Fig. 6(a), the ratio of the sensible heat flux *H*_*u*_/|*H*_*r*_| has an order of between 1 and 10 during the night-time. Therefore, the ratio of the dome diameter to the urban diameter, *D*_*d*_/*D*, has an order of 1.5 to 3.5, as is shown in Fig. 3(b). These calculated results agree well with the results of the numerical simulations and field measurements shown in Table 1. Although the ratio *D*_*d*_/*D* increases with the buoyancy frequency *N* or with the ratio of the building walls *f*_*w*_, when the other parameters are fixed, *D*_*d*_/*D* is not influenced dramatically, as can be seen in Figs 4 and 5. Increase the ratio of the building walls *f*_*w*_ leads to the increase of the convective heat transfer in the urban area and then the increase of the energy transported to the rural area from the urban area. Therefore, a larger area (i.e., a larger dome diameter) is required to release the remaining heat in the dome over the rural area. Increase the buoyancy frequency *N* results in a lower reverse height and thus reducing the flow rate between the urban area and the rural area. However, the energy transported between the urban area and the rural area keep unchanged based on Equation (2) and (3). To satisfy the energy balance, the temperature difference between the urban area and the rural area increases based on Equation (2), resulting in a lower air and surface temperature in the rural area. Therefore, a larger area is required to release the remaining heat in the dome over the rural area, because of the reduction of heat flux caused by the lower temperature in the rural area. The main parameter that determines the ratio *D*_*d*_/*D* at night is the ratio of the sensible heat flux to the absolute value of the rural heat flux, which has an order of 1 to 10, as analyzed above.

### Linearized model

Vukovich^33^ proposed a linearized model to calculate the two-dimensional urban heat dome flow at a mean condition of no background wind. The non-linear convection terms in the governing equations are replaced by friction terms, so that the governing equations can be described by linear equations. The resulting linearized equations are shown as Equations (10)–(11).

10 $$\frac{\partial u}{\partial t}+Ku=-\frac{1}{{\rho }_{0}}\frac{\partial p}{\partial x}$$

11 $$\frac{\partial w}{\partial t}+Kw=-\frac{1}{{\rho }_{0}}\frac{\partial p}{\partial z}-\frac{\rho }{{\rho }_{0}}g$$

where *u* and *w* are the horizontal and the vertical velocity component perturbations, respectively, *t* is time, *K* is the friction coefficient, *ρ*_0_ is the reference density, *p* is the pressure perturbation, *x* and *z* are the horizontal and vertical coordinates, respectively, *ρ* is the density perturbation, and *g* is the gravitational acceleration.

The stream functions are defined as $u=\partial \psi /\partial z$ and $w=-\partial \psi /\partial x$. Combine Equations (10) and (11), and integrate the stream function. The governing equation can then be written as Equation (12).

12 $$(\frac{\partial }{\partial t}+K)(\frac{{\partial }^{2}\psi }{\partial {x}^{2}}+\frac{{\partial }^{2}\psi }{\partial {z}^{2}})+\frac{\partial g\text{'}}{\partial x}=0$$

where $g\text{'}=-\rho g/{\rho }_{0}$ is the reduced gravity.

The energy conservation equation is given as Equation (13).

13 $$\frac{\partial g\text{'}}{\partial t}-{N}^{2}\frac{\partial \psi }{\partial x}=q\text{'}\,\cos (kx)H(h-z)$$

where $N=\sqrt{g/{\theta }_{0}(\partial \theta /\partial z)}$ is the buoyancy frequency, and $q\text{'}=Q\text{'}g/{\theta }_{a}$ is the heating force. *Q*′ is the perturbation heating rate. The heat flux in the urban area is assumed to be sinusoidal, and restrained below height *h*. *H*(*h*−*z*) is the Heaviside unit function. *k* = *π*/*D*, where *D* is the urban diameter, so that the heat flux at the urban edge (*x* = *D*/2) is zero.

Integrate Equation (13) into Equation (12), neglect term ${\partial }^{2}\psi /\partial {x}^{2}$, and eliminate *g*′. Equation (14) can then be obtained as follows.

14 $$(\frac{{\partial }^{2}}{\partial {t}^{2}}+K\frac{\partial }{\partial t})\frac{{\partial }^{2}\psi }{\partial {z}^{2}}+{N}^{2}\frac{{\partial }^{2}\psi }{\partial {x}^{2}}=q\text{'}k\,\sin (kx)H(h-z)$$

The boundary conditions are $\psi =0\,{\rm{at}}\,z=0\,{\rm{and}}\,t\ge 0$, $\psi =0\,{\rm{at}}\,z=Z\,{\rm{and}}\,t\ge 0$, and $\psi =\partial \psi /\partial t=0\,$ ${\rm{at}}\,0\le z\le Z\,{\rm{and}}\,t=0$. *Z* is the top boundary of the computation domain. *ψ* can be solved from Equation (14) with the corresponding boundary conditions. The results are given by Equation (15).

$$\begin{array}{c}\psi =\sum _{i=1}^{n}(-\frac{2{c}_{pi}q\text{'}}{k{N}_{2}^{2}{N}_{1}})\{1-\frac{{c}_{pi}k}{\sqrt{{c}_{pi}^{2}{k}^{2}-{(\frac{K}{2})}^{2}}}\exp (-\frac{K}{2}t)\sin [t\sqrt{{c}_{pi}^{2}{k}^{2}-{(\frac{K}{2})}^{2}}+\varphi ]\}\\ \sin (kx)[1-\,\cos (\frac{{N}_{1}h}{{c}_{pi}})]{\{{(\frac{{N}_{1}}{{N}_{2}})}^{2}h+\frac{(Z-h){\sin }^{2}(\frac{{N}_{1}h}{{c}_{pi}})}{{\sin }^{2}[\frac{{N}_{2}(h-Z)}{{c}_{pi}}]}\}}^{-1}\,\sin (\frac{{N}_{1}z}{{c}_{pi}})\,{\rm{when}}\,0\le z\le h\end{array}$$

15 $$\begin{array}{c}\psi =\sum _{i=1}^{n}(-\frac{2{c}_{pi}q\text{'}}{k{N}_{2}^{2}{N}_{1}})\{1-\frac{{c}_{pi}k}{\sqrt{{c}_{pi}^{2}{k}^{2}-{(\frac{K}{2})}^{2}}}\exp (-\frac{K}{2}t)\sin [t\sqrt{{c}_{pi}^{2}{k}^{2}-{(\frac{K}{2})}^{2}}+\varphi ]\}\\ \sin (kx)[1-\,\cos (\frac{{N}_{1}h}{{c}_{pi}})]{\{{(\frac{{N}_{1}}{{N}_{2}})}^{2}h+\frac{(Z-h){\sin }^{2}(\frac{{N}_{1}h}{{c}_{pi}})}{{\sin }^{2}[\frac{{N}_{2}(h-Z)}{{c}_{pi}}]}\}}^{-1}\,\sin (\frac{{N}_{1}h}{{c}_{pi}})\\ \sin [\frac{{N}_{2}(z-Z)}{{c}_{pi}}]{\sin }^{-1}[\frac{{N}_{2}(h-Z)}{{c}_{pi}}]\,{\rm{when}}\,h\le z\le Z\end{array}$$

where *N*_1_ and *N*_2_ are the buoyancy frequencies between 0 and *h*, and between *h* and *Z*, respectively, $\varphi =[\sqrt{{c}_{pi}^{2}{k}^{2}-{(K/2)}^{2}}/(K/2)]$, and *c*_*pi*_ is the solution for the following Equation (16).

16 $${N}_{1}\,\cot (\frac{{N}_{1}h}{{c}_{pi}})+{N}_{2}\,\cot [\frac{{N}_{2}(Z-h)}{{c}_{pi}}]=0$$

The results are analysed based on the solution given by Vukovich^33^, which is shown in Equation (15).

The horizontal velocity component *u* can be calculated through Equation (15). To calculate the horizontal velocity profiles at a different location, the urban parameters are assigned common values. The boundary layer height, $h=-|x|/20+1000$(m), is assumed to change linearly. The origin is located at the urban centre. Urban diameter *D* is assumed to be 20 km. Friction coefficient *K* is 1 × 10^−4^ s^−1^. Time *t* is 1800 s. Heating rate *Q*′ is 1/1800 K s^−1^. Top boundary height *Z* is 10 km. Flow boundary layer height at urban centre *h*_*0*_ is 1 km. Buoyancy frequency in the lower level *N*_*1*_ and in the upper level *N*_*2*_ are same, which is 0.0179 s^−1^. Reference temperature *θ*_0_ is 300 K.

**Figure 7:**
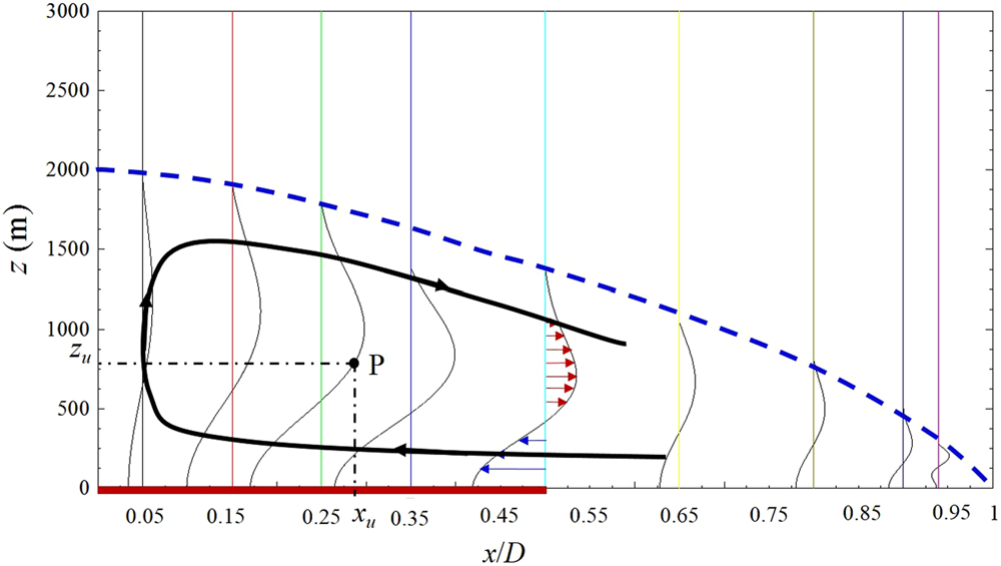
Horizontal velocity profiles as calculated from Equation (15). The velocity field is symmetrical about the urban centre (*x* = 0), so that only the right half of the velocity field is drawn in the figure. The profiles at different locations (*x* coordinates) are marked by the vertical solid lines. The dashed curve is used to mark the urban heat dome flow perimeter. The thick solid curve with arrows illustrates the typical streamline in the urban heat dome flow. The thick red line on the horizontal axis marks the urban location (between *x* = 0 and *x* = 10). The velocity values on the different curves are rescaled in this figure, according to the different vertical lines that mark the positions. The original velocity value on one specific velocity profile curve can be calculated for the following example. Point P represents the horizontal velocity, $u=({x}_{u}-7)/2$ (m s^−1^), at position (7 km, *z*_*u*_).

Equations (15) and (16) are solved numerically by MATLAB. The results are shown in Fig. 7.

The results shown in Fig. 7 indicate that the horizontal velocity decreases to a small value toward the position *x* = 20 km. This result suggests that the dome’s horizontal diameter is about two times that of the urban diameter, i.e., *D*_*d*_/*D* has an order of 2. The relatively large vertical size might result from the neglect of term ${\partial }^{2}\psi /\partial {x}^{2}$ (which does not hold for the urban centre upward flow region), and from the accuracy of the estimation of parameter values, such as the values of the friction coefficient, the buoyancy frequency, and the heating rate. Nevertheless, the velocity field is qualitatively meaningful. The horizontal extent of the urban heat dome flow can also be supported directly by the solution from Equation (15), as can be verified easily by noting that when *x* = *D*, the term sin(*kx*) = 0, which results in the velocity being zero. This finding shows that the urban heat dome flow’s horizontal edge is reached when *x* = *D*, which indicates that *D*_*d*_/*D* = 2 according to this model. However, it should be noted that in this linearized model, the ratio *D*_*d*_/*D* does not change with other parameters such as the buoyancy frequency, urban sensible heat flux, or rural sensible heat flux. This consistency in the *D*_*d*_/*D* ratio is maintained because the term sin(*kx*) = 0 is contained in the solution when *x* = *D*, which eliminates the influence of other parameters. Therefore, the linearized model has limitations in analyzing the urban heat dome flow and the relevant influencing factors.

In the linearized model, the governing equations for the fluid flow are solved by using perturbation method. The flow field is solved based on the assumption that the buoyancy force is balanced by the friction. In the energy balance model, the governing equations for the heat transfer in the urban area and in the rural area are solved to get the horizontal extent of the heat dome. The radiative heat transfer and convection heat transfer are considered and modelled over the urban and rural area in the energy balance method, which enables to analyse and quantify the influencing factors on the urban heat dome flow.

### Determining daytime dome horizontal extent

An estimated scale of the ratio *D*_*d*_/*D* can be obtained by comparing the turbulent convective velocity scale and the velocity scale of the urban heat dome flow. According to Deardorff^34^, the maximum turbulent velocity scale in the convective boundary layer can be described by ${\sigma }_{w}/w\ast =0.61\pm 0.02$, where $w\ast ={(g\beta {z}_{ir}{H}_{r})}^{1/3}$ is the turbulent convective velocity scale, and *z*_*ir*_ is the mixing height in the rural area. *β* and *g* are the thermal expansion rate and the gravity acceleration, respectively. The maximum mean radial velocity of ${U}_{D/2}/W\ast =1.455$ at the urban edge is given by Hidalgo *et al*.^21^, where $W\ast ={[g\beta {z}_{iu}({H}_{u}-{H}_{r})]}^{1/3}$ is the daytime radial velocity scale of the urban heat dome flow at the urban-rural interface, and *z*_*iu*_ is the mixing height at the urban centre. Lu^35^ proposed the hydrostatic convection model, where the difference in pressure between the urban area and the ambient region is used to calculate the radial velocity scale. The hydrostatic convection model is proven to work well for predicting the radial velocity at the urban-rural interface, which suggests that the urban heat dome flow in the lower convergent inflow region can be assumed as a potential sink flow, and the upper divergent outflow region can be assumed as a potential source flow. The intensity of the potential flow can be estimated by the flow rate at the urban edge, and the virtual source or sink is assumed to lie at the urban centre. Therefore, the radial velocity should decrease with the increase of distance between the study location and the urban centre. The following Equation (17) is given in accordance with the mass conservation and the velocity scales obtained from Hidalgo *et al*.^21^.

17 $${U}_{R}=\frac{D}{{D}_{d}}{U}_{D/2}=\frac{D}{{D}_{d}}\times 1.455{[g\beta {z}_{iu}({H}_{u}-{H}_{r})]}^{1/3}$$

where *U*_*R*_ and *U*_*D/2*_ are the velocity scales at the dome edge and at the urban edge, respectively. When the mean radial velocity has the same order as the convective velocity scale, the horizontal extent of the dome is restricted during the daytime, so that the following Equation (18) can be obtained.

18 $${U}_{R}\simeq {\sigma }_{w}=0.61{(g\beta {z}_{ir}{H}_{r})}^{1/3}$$

Based on the results obtained by Wang^36^, *z*_*ir*_ is about 2/3 of the *z*_*iu*_. As shown in Fig. 3(b), *H*_*u*_ ranges from 73 W m^−2^ (17:00) to 202 W m^−2^ (13:00), and *H*_*r*_ ranges from 36 W m^−2^ (17:00) to 125 W m^−2^ (12:00) during the daytime. The ratio of *H*_*u*_ to *H*_*r*_ is about 1.4 to 2.8, based on the data shown in Fig. 3(b). Therefore, *D*_*d*_/*D* ~ 2.0–3.3 is obtained for the daytime situation, with consideration of the influence of convective turbulence in the rural area after applying Equations (17) and (18).

## Discussion

According to the energy balance model (Section 2.1), the linearized model (Section 2.2), the daytime situation analysis (Section 2.3), the field measurement (summarized in Table 1), and the CFD simulation (summarized in Table 1), the ratio *D*_*d*_/*D* ranges from 1.5 (from the energy balance model) to 4 (from Lemonsu and Masson^15^, as shown in Table 1). The results obtained by the energy balance model agree well with previous field measurements^16,19^ and with previous numerical simulation results^14,15,19^, as summarized in Table 1. Note that the energy balance model only applies to the night-time condition. During the day, convective eddies exist in the rural area, and the urban dome is influenced the convective turbulence in the rural area. The mixing height and the average temperature in the urban heat dome flow also change over time. The energy balance model does not apply during the daytime, because both the urban and the rural areas supply positive heat flux to the urban heat dome flow. The urban heat dome flow may not exist for a certain period during the daytime because of the urban cool island effect^37^. However, when the urban heat dome flow does exist during the daytime, the horizontal extent of that flow can be estimated by using the turbulent convective velocity scale and the velocity scale of the urban heat dome flow, as analysed in Section 2.3. The characteristics of the urban heat dome flow in the drylands would also be different from that in the region with abundant vegetation, due to the difference of the background rural area properties^23,24^, i.e., sensible heat flux in the rural area.

The relationship between the urban diameter and the horizontal extent of the urban heat dome flow has important implications for the transportation of pollutants at the city and regional scale. Assuming that the projection of the urban heat dome flow on the ground creates a circular region (defined as the influence region), this study’s results imply that the diameter of the influence region can be as large as four times the urban diameter. Therefore, the pollutant sources within the influence region can be transported across the urban area when the urban heat dome flow is formed. The velocity scale of the urban heat dome flow is in the order of 2 m s^−1^ ^10,19^. Assuming that the urban diameter is in the range of 30 km, the diameter of the influence domain would be in the range of 45 to 120 km. If a pollutant source is located at the edge of the influence domain (i.e., if the distance between the pollutant source and the urban edge is between 7.5 km and 45 km), then the time required for the transport of the pollutants from the source to the urban edge would be between 1 and 6 hours (calculated using the distance divided by the velocity). This finding implies that pollutants can be gathered by the urban heat dome flow across the urban area within one night. Two adjacent cities, for example city A and city B, can influence each other through the urban heat dome flow. Assuming that both city A and city B have the same diameters of 30 km each, and they both have the same urban heat island intensity under the same background conditions, then these two cities can send their pollutants to each other if the distance from the edge of city A to the edge of city B is between 15 and 90 km (as calculated based on the influence domain of the two urban heat dome flows). Hence, the distance between two cities becomes an important parameter for regional air pollution control. Take the city cluster in Beijing-Tianjin-Hebei region for example, the city diameter of Beijing (BJ), Langfang (LF) and Tianjin (TJ) is in the order of 40 km, 10 km and 30 km respectively, assuming the cities are of circular shape. The city of LF locates between BJ and TJ. The distance between BJ and LF, i.e. from the centre of BJ to the centre of LF, and that between LF and TJ are in the order of 45 km and 60 km. The diameter of the heat dome flows generated by BJ, LF and TJ are thus in the order of 60–160 km, 15–40 km and 45–120 km respectively, according to our results. Therefore, the three heat domes generated by BJ, LF and TJ can develop independently without interaction when the diameters of the heat domes are in the smallest range (60 km, 15 km and 45 km for BJ, LF and TJ respectively). Although the distance between BJ and TJ is as large as 105 km, the heat dome generated by BJ and by TJ can interact with each other when the diameters of the heat domes are in the largest range (160 km, 40 km and 120 km for BJ, LF and TJ respectively). In this situation, LF is totally submerged in the heat dome generated by BJ.
